# Supplementary material for: Bioinspired Dopamine and N-Oxide-Based Zwitterionic Polymer Brushes for Fouling Resistance Surfaces
Source: Polymers (Basel). 2024 Jun 9;16(12):1634. doi: 10.3390/polym16121634 (PMC11207554; doi:10.3390/polym16121634)
Supplement: Supplementary file 1 [file polymers-16-01634-s001.zip › polymers-3020380-supplementary.pdf]

Bioinspired Dopamine and N-Oxide-Based Zwitterionic Polymer Brushes for Fouling Resistance Surfaces

Zhen Zhou [1] and Qinghong Shi [1,2,*]

[1] Department of Biochemical Engineering, School of Chemical Engineering and Technology, Tianjin University, Tianjin 300350, China; zhou_zhen@tju.edu.cn
[2] Key Laboratory of Systems Bioengineering (Ministry of Education), Tianjin University, Tianjin 300072, China
* Correspondence: qhshi@tju.edu.cn

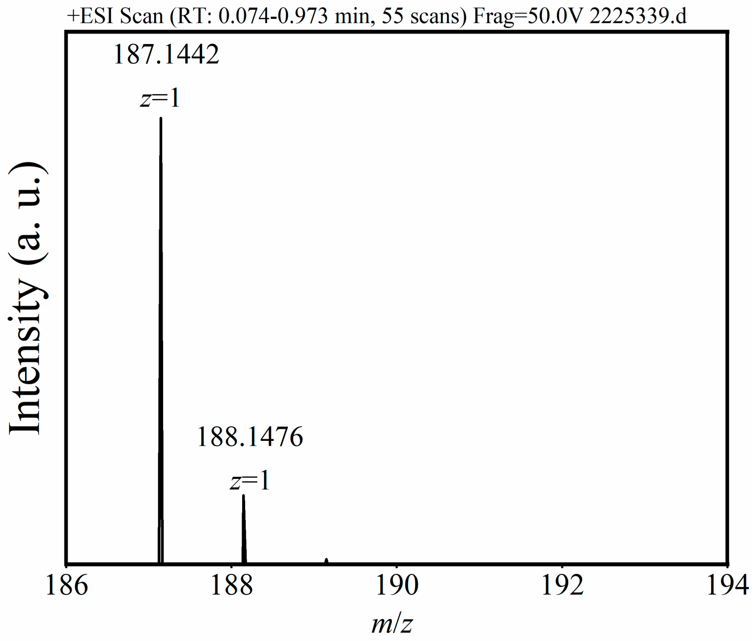

Figure S1 HRMS spectrum of MADMPAO.

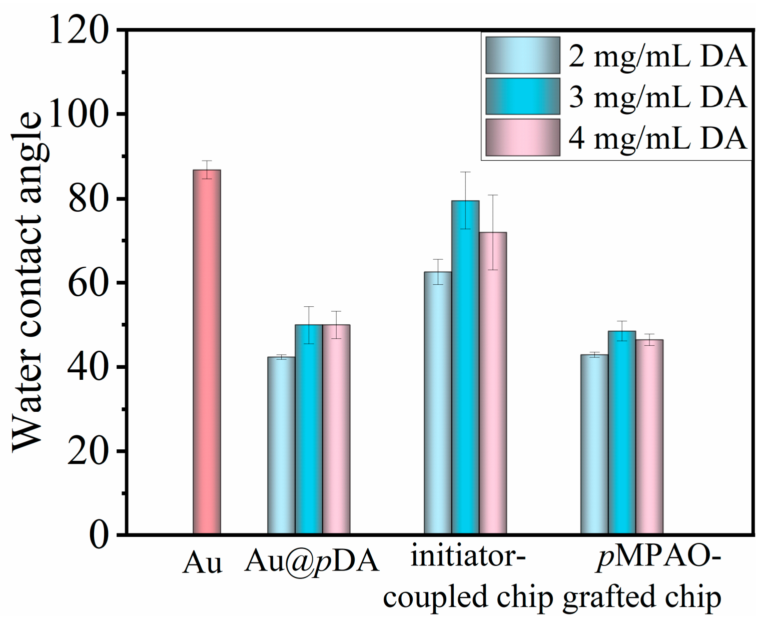

Figure S2 Water contact angles of bare gold, *p*DA-coated, initiator-coupled and *p*MPAO-grafted chips.

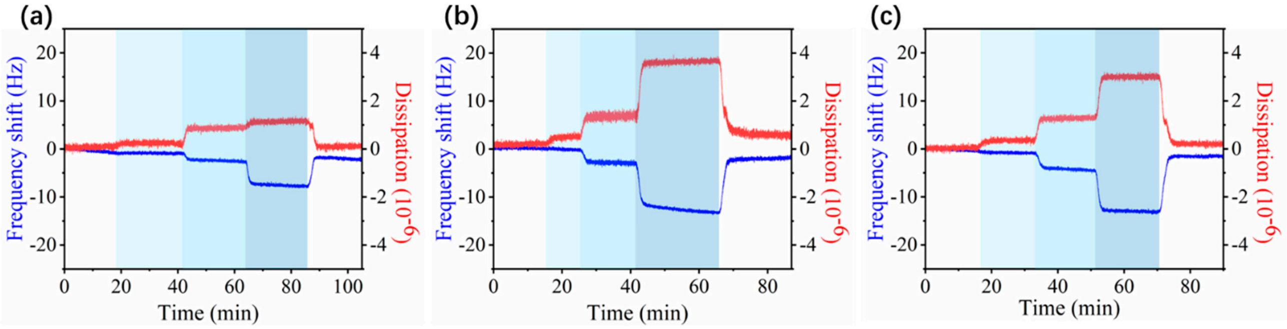

Figure S3 Monitoring of frequency and dissipation shift versus time on initiator-coupled chips in the presence of NaCl (a), Na_2SO_4 (b) and $MgCl_2$ (c) with different concentrations (1.0, 10, and 100 mmol/L).

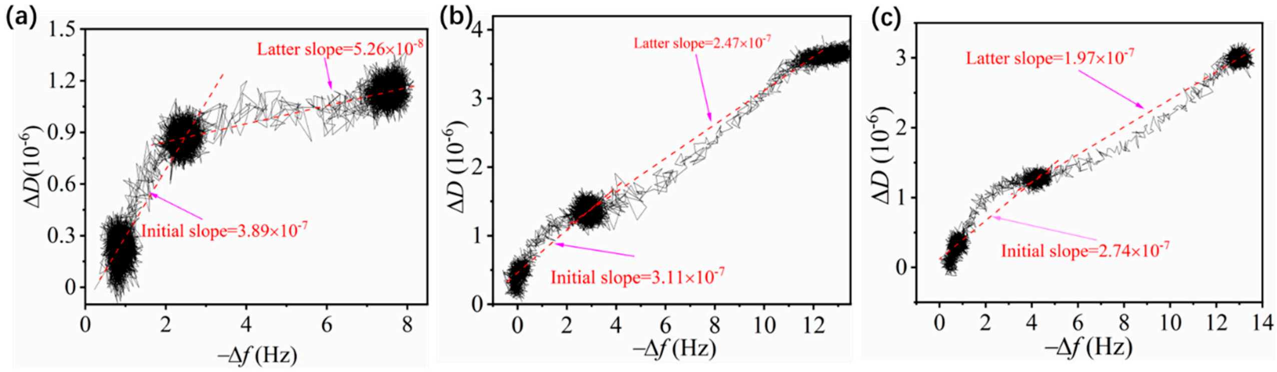

Figure S4 Monitoring of dissipation shift versus frequency on initiator-coupled chips in the presence of NaCl (a), Na_2SO_4 (b) and $MgCl_2$ (c) with different concentrations (1.0, 10, and 100 mmol/L).

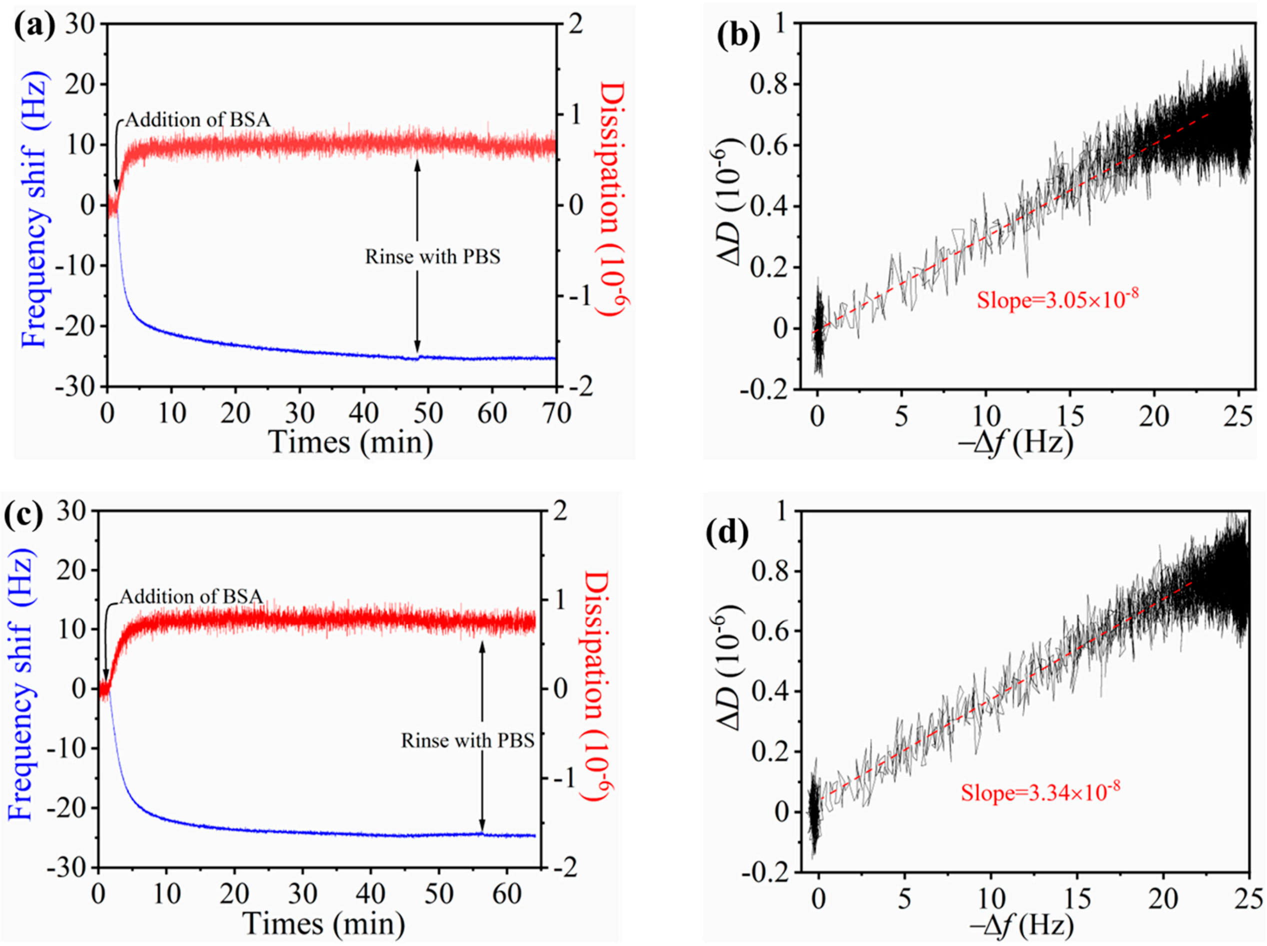

Figure S5 QCM measurement of BSA adsorption on bare gold and Au@*p*DA-2 chips. (a) time resolved profile of Δf and ΔD on bare gold chip, (b) $D-f$ plot on bare gold chip, (c) time resolved profile of Δf and ΔD on Au@*p*DA-2 chips and (d) $D-f$ plot on Au@*p*DA-2 chips.

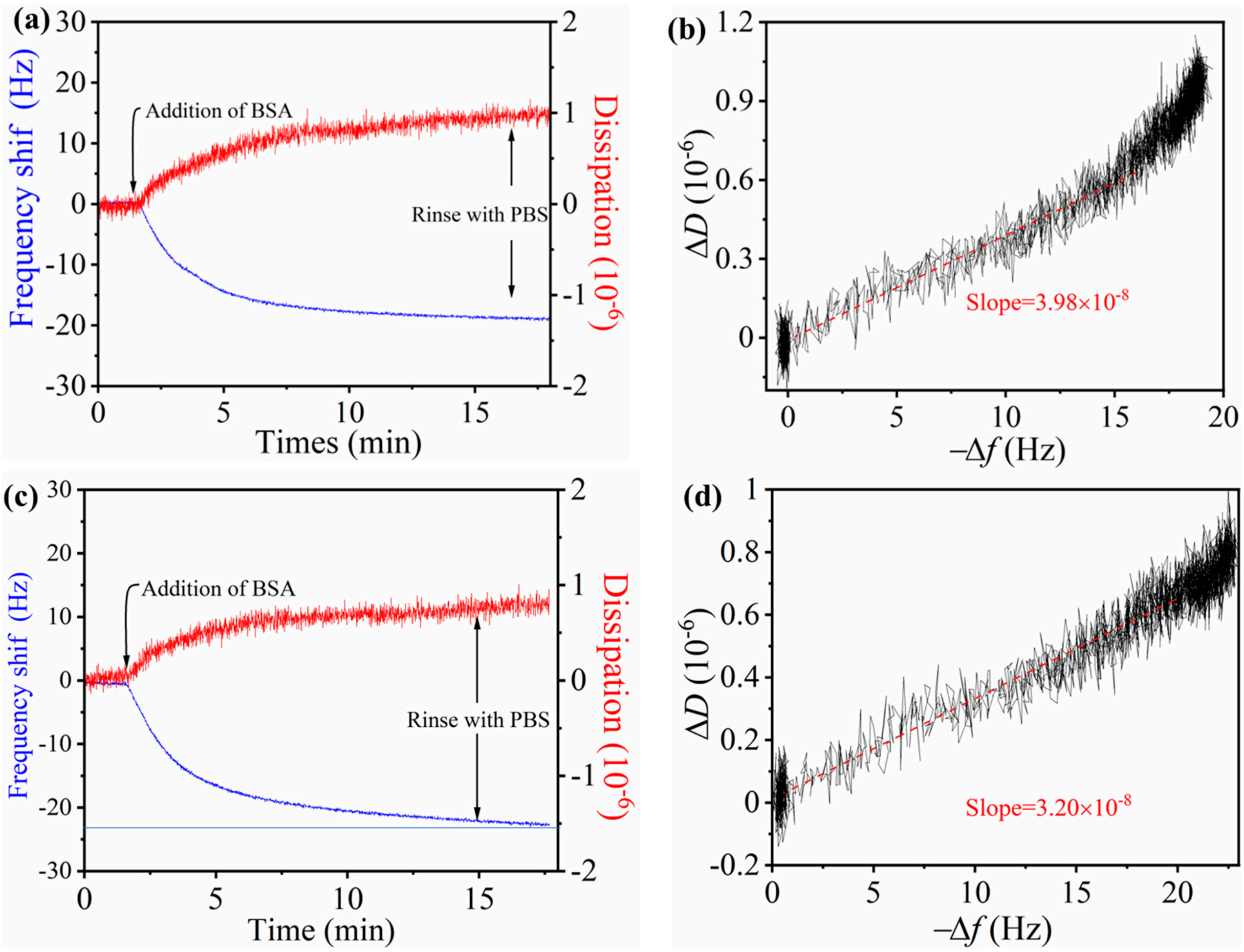

Figure S6 QCM measurement of BSA adsorption on initiator-coupled chips. (a) time resolved profile of Δf and ΔD on Au@*p*DA-3-Br chip, (b) $D-f$ plot on Au@*p*DA-3-Br chip, (c) time resolved profile of Δf and ΔD on Au@*p*DA-4-Br chip and (d) $D-f$ plot on Au@*p*DA-4-Br chip
